# Characterizing diabetic cardiomyopathy: baseline results from the ARISE-HF trial

**DOI:** 10.1186/s12933-024-02135-z

**Published:** 2024-02-01

**Authors:** James L. Januzzi, Stefano Del Prato, Julio Rosenstock, Javed Butler, Justin Ezekowitz, Nasrien E. Ibrahim, Carolyn S.P. Lam, Thomas Marwick, W. H. Wilson Tang, Yuxi Liu, Reza Mohebi, Alessia Urbinati, Faiez Zannad, Riccardo Perfetti

**Affiliations:** 1 Heart Failure Trials, Baim Institute for Clinical Research, Boston, MA USA; 2 Cardiology Division, Massachusetts General Hospital, Harvard Medical School, 55 Fruit Street, 02114 Boston, MA USA; 3 Interdisciplinary Research Center ‘Health Science’, Sant’Anna School of Advanced Studies, Pisa, Italy; 4 Velocity Clinical Research at Medical City and University of Texas, Southwestern Medical Center, Dallas, TX USA; 5 Baylor Scott and White Research Institute, , Dallas, TX USA; 6 University of Mississippi, Jackson, MS USA; 7 Canadian VIGOUR Centre, University of Alberta, Edmonton, AB Canada; 8 Cardiology Division, Brigham and Women’s Hospital, Harvard Medical School, Boston, MA USA; 9 National Heart Centre Singapore and Duke-National University of Singapore, Singapore, Singapore; 10 Baker Heart and Diabetes Institute, Melbourne, Australia; 11 Menzies Institute for Medical Research, Hobart, Australia; 12 Department of Cardiovascular Medicine, Heart Vascular and Thoracic Institute, Cleveland Clinic, Cleveland, OH USA; 13 Applied Therapeutics Inc, New York, NY USA; 14 Université de Lorraine, CIC Inserm and CHRU Nancy, Lorraine, France

**Keywords:** Diabetes, Cardiomyopathy, Heart failure

## Abstract

**Background:**

Diabetic cardiomyopathy (DbCM) is a form of Stage B heart failure (HF) at high risk for progression to overt disease. Using baseline characteristics of study participants from the Aldose Reductase Inhibition for Stabilization of Exercise Capacity in Heart Failure (ARISE-HF) Trial we sought to characterize clinical characteristics of individuals with findings consistent with DbCM.

**Methods:**

Among study participants meeting inclusion criteria, clinical characteristics, laboratory testing, imaging, Kansas City Cardiomyopathy Questionnaire (KCCQ), Physical Activity Scale of the Elderly (PASE) and cardiopulmonary exercise testing (CPET) results were tabulated. Cluster phenogroups were identified.

**Results:**

Among 691 study participants (mean age 67.4 years; 50% were female), mean duration of type 2 diabetes mellitus (T2DM) was 14.5 years. The median (Q1, Q3) N-terminal pro-B type natriuretic peptide and high sensitivity cardiac troponin T were 71 (35, 135) ng/L and 9 [6, 12] ng/L. The most common echocardiographic abnormalities were reduced global longitudinal strain in 25.3% and impaired diastolic relaxation in 17.7%. Despite rather well-preserved KCCQ scores the average PASE score was markedly impaired at 155 accompanied by an average maximal oxygen consumption of 15.7 mL/Kg/minute on CPET. In K-means clustering, 4 phenogroups were identified including a higher-risk group with more advanced age, greater elevation of cardiac biomarkers, and more prevalent evidence for diastolic dysfunction and left ventricular hypertrophy.

**Conclusions:**

Baseline data from the ARISE-HF Trial provide clinical characterization of individuals with T2DM and features of stage B HF, and may help clarify the diagnosis of DbCM.

**Trial Registration:**

ARISE-HF, NCT04083339.

**Supplementary Information:**

The online version contains supplementary material available at 10.1186/s12933-024-02135-z.

## Background

First described in postmortem studies by Rubler et al. in 1972 [1], diabetic cardiomyopathy (DbCM) is an increasingly recognized but insufficiently studied complication of diabetes leading to heart muscle disease [2–4]. At its early stages, DbCM is considered a form of Stage B heart failure (HF), defined as structural heart disease without past or present overt HF [5, 6] and is a risk for progression to symptomatic stages of the diagnosis. Presumably due to significant chronic hyperglycemia, DbCM may occur in the absence of other causes of cardiac dysfunction such as coronary artery disease (CAD), clinically relevant arrhythmias, severe valvular heart disease, and uncontrolled blood pressure, however each of these may accelerate progression to later stage HF. DbCM may occur in any form of diabetes mellitus, however most data regarding the diagnosis in patients focuses on type 2 diabetes mellitus (T2DM).

Histologically, DbCM is characterized by a non-specific pattern of cardiomyocyte hypertrophy, accompanied by interstitial inflammation and fibrosis [2–4] and may be accompanied by cardiovascular autonomic neuropathy [7]. Clinically, tissue damage from DbCM may result in elevated cardiac biomarkers [4, 8, 9], impaired myocardial relaxation, and abnormal systolic function [10] with resulting elevated filling pressures; in sum, this creates a risk for progression to overt HF. Significant functional limitation on exercise testing is commonly described in individuals with DbCM [11], wrongly attributed to “low endurance or laziness”, but the diagnosis is clinically heterogeneous and a large, well-defined cohort might provide more information on the clinical features. Indeed a deeper characterization of DbCM, based on bio-humoral, cardiopulmonary, and echocardiographic testing has been advocated, as it might predict transition to and progression of HF; it may also identify treatment opportunities to improve cardiac function in affected individuals [12–15].

At the tissue level, activation of several deleterious pathways has been implicated in the processes leading to myocardial injury with fibrosis in those with DbCM [16–22]. Among them is hyperactivation of the polyol pathway [18–22], thought to be one of the primary mechanisms contributing to the development of DbCM. Aldose reductase catalyzes the first and rate-limiting step in the polyol pathway, and its inhibition has been shown to possibly reduce diabetic complications including DbCM [23]. The Aldose Reductase Inhibition for Stabilization of Exercise Capacity in Heart Failure (ARISE-HF) Trial [24] is a global phase 3 randomized study evaluating the safety and efficacy of two doses of a novel highly-selective aldose reductase inhibitor (AT-001) versus placebo to improve or prevent decline in functional capacity in individuals with DbCM. We utilized the resources of the ARISE-HF data to define the baseline characteristics of individuals with DbCM, including an evaluation of the presence and extent of abnormal cardiac biomarkers, cardiac function, and exercise impairment in the study participants. We additionally explored whether definable clusters of phenotypes might be present among individuals with DbCM.

## Methods

The rationale and design of the ARISE-HF trial (NCT04083339) has been previously published [24]. In brief, this global study is a Phase 3, randomized, placebo-controlled study to assess the efficacy of AT-001 compared with placebo for preservation or improvement of exercise capacity as measured by peak oxygen uptake (peak VO2) during a cardiopulmonary exercise testing (CPET) as well as assess the safety and tolerability of AT-001 over the long-term in adult study participants with T2DM and prevalent features of Stage B HF and without significant hyperglycemia, no evidence of ischemic heart disease, uncontrolled hypertension or other causes of HF. Stage B HF was defined by elevated cardiac biomarkers and/or the presence of cardiac structural/functional abnormalities. Study participants were required to have evidence of impaired functional capacity defined as peak VO2 uptake below 75% of predicted normal on a CPET. Notably, because of regulatory expectations, study participants were required to have well-controlled T2DM at the time of enrollment; in a similar fashion, blood pressure was required to be well-controlled. The key inclusion criteria for the trial have been previously published [24] and are listed in Supplemental Table 1.

Baseline demographics and past medical history were extracted from the electronic case report form. Frequency of medications use for diabetes and other medical conditions was recorded. Laboratory investigations included measurement of N-terminal pro-B type natriuretic peptide (NT-proBNP) and high sensitivity cardiac troponin T (hs-cTnT). Additionally, hemoglobin A1c (HbA1c), blood hemoglobin, as well as measures of kidney function (estimated glomerular filtration rate [eGFR] using the race-free CKD-Epi equation) and albumin/creatinine ratio were measured, as were serum lipids.

A 2-dimensional echocardiogram was performed and interpreted by a core lab blinded to treatment assignment. Variables evaluated included measures of systolic function (left ventricular ejection fraction [LVEF] and global longitudinal strain [GLS]), diastolic function (the ratio of early transmitral Doppler flow [E] over the early septal annular velocity [e’]), left atrial volume and left ventricular mass both indexed for body surface area (LAVi and LVMi respectively) and estimated pulmonary artery pressures (expressed as right ventricular systolic pressure; RVSP). An abnormal LVEF was an exclusion for the trial. Abnormalities in the remaining variables could be as follows: GLS >-16%; E/e’ ≥ 13; LAVi > 34 mL/m^2^; LVMi ≥ 115 g/m^2^ in men and ≥ 95 g/m^2^ in women; RVSP > 35 mm Hg.

Measures of health status and activity were taken at baseline. These include the 23-question Kansas City Cardiomyopathy Questionnaire (KCCQ-23; modified to consider the study population does not have overt HF) as well as the Physical Activity Score for the Elderly (PASE) score (range from 0 to 793) [25]; this score was developed to provide an instrument to categorize physical activity levels during a 7 day look-back period, and is calculated using 12 questions regarding frequency and duration of activity multiplied by time spend in various activities and summing the overall scores. Higher scores indicate higher levels of physical activity with normative values dependent on age and sex categories.

Lastly CPET results were tabulated; for the purposes of this report, the duration of exercise, the peak VO_2_, the slope of the ratio of minute ventilation/carbon dioxide production (VE/VCO_2_ slope; abnormal is > 32.8) and peak respiratory exchange rate (RER; values > 1.05 indicate adequate test performance) are detailed.

To seek whether phenogroups within study participants might be identified, we employed unsupervised machine learning using K-means clustering to uncover inherent patterns within the dataset, allowing us to identify subpopulations of patients with similar characteristics. This method was applied to partition the cohort of patients leveraging a multidimensional set of clinical variables that included age, sex, race, BMI, medical history, smoking status and baseline echocardiographic measurements. To elucidate the differences between the identified phenogroups, we used the chi-square test to assess the significance of differences between the clustering groups for categorical variables. The Wilcoxon rank-sum test was utilized to compare the medians between groups for non-normally distributed continuous variables and t-test for continuous variables that followed a normal distribution. To identify variables of greatest weight within each group, the centroid for variables within each cluster was determined and then differences among these cluster centroids for each variable were compared.

All statistical analyses were performed using the R version 4.2.3 (R Foundation for Statistical Computing, Vienna, Austria. URL: https://www.R-project.org/). *P* values are two-sided with values < 0.05 considered statistically significant.

### Data and resource availability

The datasets generated during and/or analyzed during the current study are not publicly available due to the ongoing ARISE-HF Trial.

## Results

Between September of 2019 and October of 2022, 691 study participants meeting inclusion and exclusion criteria were enrolled in 62 sites as shown in Supplemental Fig. 1.

Baseline characteristics of the study participants are detailed in Table 1. Supplemental Table 2 shows baseline characteristics between those included versus those not included in the trial, which demonstrates no significant differences among the available variables analyzed. Among those included in the study, the average age was 67.4 years and 50% were female. Notably, nearly 22% were of Hispanic ethnicity. Although 7.1% of the study participants were of Black race, proportionally among those enrolled in the United States, this percentage was 12.8%.

**Table 1:** Baseline characteristics in the ARISE HF Trial

n	691
Age, years, mean (SD)	67.4 (7.2)
Female, N (%)	348 (50.4)
**Race, N (%)**	
White, non-Hispanic	432 (62.5)
Hispanic	151 (21.9)
Black	42 (6.1)
Asian	56 (8.1)
American Indian or Alaskan Native	4 (0.6)
Other	6 (0.9)
Body-mass index, kg/m^2^, mean (SD)	30.6 (4.57)
**Smoking Status, N (%)**	
Current Smoker	64 (9.3)
Previous Smoker	240 (34.7)
Never	387 (56.0)
**Vital signs at screening**	
Systolic blood pressure, mmHg, mean (SD)	129 (11)
Diastolic blood pressure, mmHg, mean (SD)	76 (8)
Heart rate, beats/minute, mean (SD)	69 (11)
**Medical history, N (%)**	
Hypertension	523 (75.7)
Dyslipidemia	117 (16.9)
Duration of T2DM, years, mean (SD)	14.5 (10.1)
**Concomitant medications, N (%)**	
Statins	558 (80.8)
Angiotensin converting enzyme inhibitor or angiotensin II receptor blocker	519 (75.1)
Beta blocker	161 (23.3)
Mineralocorticoid receptor antagonist	21 (3.0)
Hydrochlorothiazide	133 (19.2)
Sodium/glucose cotransporter 2 inhibitor	221 (32.0)
Glucagon-like peptide-1 receptor agonist	173 (25.0)
Metformin	512 (74.1)
Insulin	188 (27.2)
Sulfonylurea	160 (23.2)
Dipeptidylpeptidase-4 inhibitor	85 (12.3)

SD denotes: standard deviation; Kg/m^2^ denotes: kilogram per meter squared; mmHg denotes: millimeters of mercury; T2DM denotes: type 2 diabetes mellitus;

Most the study participants had a history of hypertension but, as per protocol, were well-managed with average blood pressures on enrollment below the recommended target for individuals with diabetes. Most were receiving an angiotensin converting enzyme inhibitor or angiotensin II receptor blocker (75.1%) while relatively fewer received other antihypertensives.Most (80.8%) were treated with a lipid lowering agent.

Patients enrolled had a duration of T2DM of 14.5 years, with excellent glycemic control at baseline (HbA1c of 6.98%), as required per protocol. Metformin was the most utilized hypoglycemic agent (74.1%), however use of both sodium/glucose co-transporter 2 (SGLT2) inhibitor and glucagon-like peptide-1 receptor agonist (GLP-1RA) use was notable at 32.0% and 25.0%, respectively. Insulin use was present in 27.2%, while sulfonylurea and dipeptidylpeptidase-4 (DPP4) inhibitors were less commonly employed at 23.0% and 12.3%.

From a laboratory testing perspective (Table 2), the median (Q1, Q3) NT-proBNP was 71 (35, 135) ng/L; 26.6% had an NT-proBNP at or above 125 ng/L. The median (Q1, Q3) hs-cTnT was 9 [6, 12] ng/L with 20.4% of study participants possessing values at or above the 99th percentile hs-cTnT concentration for a healthy population. As defined by inclusion/exclusion criteria, kidney function was relatively preserved (eGFR of 80.5 mL/min/1.73m^2^; 24.7% with an eGFR < 60 mL/min/1.73m^2^) and there was a low prevalence of microalbuminuria. Lipids were well-managed.

**Table 2:** Results of baseline testing

n	691
**Laboratory tests, median (Q1, Q3) unless specified**	
N-terminal pro-B type natriuretic peptide, ng/L	71 (35, 135)
High sensitivity cardiac troponin T, ng/L	9 (6, 12)
Hemoglobin A1c, mean ± SD	6.98 (0.79)
Hemoglobin, g/L, mean ± SD	13.7 (1.4)
CKD-Epi, eGFR, mL/min/1.73m^2^, mean ± SD	80.5 (16.3)
Albumin/Creatinine	15 (8, 41)
Total cholesterol, mg/dL	154 (133, 183)
High density lipoprotein cholesterol, mg/dL	47 (39, 58)
Low density lipoprotein cholesterol, mg/dL	72 (55, 97)
Triglycerides, mg/dL	129 (101, 182)
**Echocardiographic measurements, median (Q1, Q3)**	
E/e’	9.40 (7.80, 11.90)
Global longitudinal strain, %	-17.7 (-19.5, -15.2)
Left atrial volume index, mL/m^2^	23.4 (19.1, 28.9)
Left ventricular ejection fraction, %	62 (59, 66)
Left ventricular mass index, g/m^2^	74 (64, 88)
Right ventricular systolic pressure, mmHg	23 (19, 28)
**Abnormal echocardiogram, N, %**	
Global longitudinal strain > -16%	175 (25.3)
E/e’ ≥ 13	122 (17.7)
Left atrial volume index > 34 mL/m^2^	82 (11.9)
Left ventricular mass index ≥ 115 g/m^2^ in men and ≥ 95 g/m^2^ in women	82 (11.9)
Right ventricular systolic pressure > 35 mmHg	27 (3.9)

ng/L denotes: nanograms per liter; Q denotes: quartile; g/L denotes: grams per liter; mg/dL denotes: milligrams per deciliter; g/m^2^ denotes: grams per meter squared; mmHg denotes: millimeters of mercury; E/e’ denotes: ratio of early transmitral Doppler velocity/early diastolic septal annular velocity

Among echocardiographic abnormalities evaluated at enrollment, the most common were abnormal GLS (in 25.3%) and impaired diastolic relaxation in 17.7%. Other abnormalities, including increase in LAVi or LVMi or presence of pulmonary hypertension were less frequently observed but nonetheless noteworthy.

Despite rather well-preserved KCCQ scores (Table 3) with total symptom score (TSS), clinical summary score (CSS) and overall summary score (OSS) results above 90 points, the average PASE score was 155, indicating approximately half of the study participants had significantly reduced physical activity. This is echoed by the results of the CPET (Table 4), with an average duration of slightly less than 10 min, with marked impairment of physical capacity: the average VO2max was only 15.7 mL/Kg/minute (below the 5th percentile of normal for the average age of the study) and nearly half had an abnormal VE/VCO2 slope.

**Table 3:** Results of questionnaires at baseline. Results are mean (standard deviation)

Questionnaires, all mean (SD)	
KCCQ physical limitation score	89 (16)
KCCQ symptom stability score	51 (10)
KCCQ symptom burden score	92 (14)
KCCQ self-efficacy score	85 (23)
KCCQ quality of life score	87 (20)
KCCQ social limitation score	92 (17)
KCCQ total symptom score	92 (14)
KCCQ clinical summary score	91 (14)
KCCQ overall summary score	90 (15)
PASE score	155 (90)

SD denotes: standard deviation; KCCQ denotes: Kansas City Cardiomyopathy Questionnaire; PASE denotes: physical activity score in the elderly

**Table 4:** Abbreviated results from baseline cardiopulmonary exercise testing. Results are mean (SD)

CPET	
Duration of CPET test, minutes	9.75 (2.40)
Peak respiratory exchange rate	1.18 (0.10)
Peak VO_2_, mL/kg/min	15.7 (3.8)
VE/VCO_2_ slope	31.2 (5.4)

SD denotes: standard deviation; CPET denotes: cardiopulmonary exercise test; VO_2_ denotes: oxygen consumption; mL/Kg/min denotes: milliliters per kilogram per minute; VE/VCO_2_ slope denotes: slope of minute ventilation/carbon dioxide production

Lastly, with K-means clustering using baseline characteristics, 4 distinct phenogroups with relatively similar cluster sizes were identified (Table 5). The 4 groups had different dominant variables; these included younger age with higher blood pressures (Cluster 1), lower BMI and lower blood pressures (Cluster 2), lower LAVi and LVMi (Cluster 3) and oldest age with higher E/e’, LAVi and LVMi (Cluster 4). Notably, besides these features, Cluster 4 also had the highest proportion of Hispanic participants, and had relatively higher NT-proBNP and hs-cTnT concentrations. It also had relatively worse kidney function relative to the other groups. Importantly, across all groups, the KCCQ was strikingly similar; the PASE score was low across all 4 groups, but Cluster 4 had among the lowest activity scores at 139. Lastly, across all 4 phenogroups exercise capacity (as evidenced by performance on the CPET) was very similar.

**Table 5:** Results of cluster analysis of patients with diabetic cardiomyopathy. 4 discrete phenogroups were identified. The 4 groups had different dominant variables; these included younger age with higher blood pressures (Cluster 1), lower BMI and lower blood pressures (Cluster 2), less diastolic dysfunction and less cardiac remodeling (Cluster 3) and oldest age with worse diastolic dysfunction, cardiac remodeling and higher biomarkers Cluster 4)

	Cluster 1 “Younger Hypertensive”	Cluster 2 “Lower BMI and lower blood pressures”	Cluster 3 “Less LA and LV remodeling”	Cluster 4 “Older with high-risk features”	*p*
n	229	140	228	94	
AGE, mean (SD)	66.2 (7.1)	67.1 (7.7)	68.2 (7.0)	68.5 (6.7)	0.02
FEMALE (%)	110 (48.0)	77 (55.0)	114 (50.0)	47 (50.0)	0.63
RACE (%)					0.03
White	142 (62.0)	83 (59.3)	153 (67.1)	54 (57.4)	
Hispanic	50 (21.8)	26 (18.6)	47 (20.6)	28 (29.8)	
Asian	24 (10.5)	14 (10.0)	16 (7.0)	2 (2.1)	
Black	10 (4.4)	13 (9.3)	11 (4.8)	8 (8.5)	
American Indian or Alaskan Native	1 (0.4)	3 (2.1)	0 (0.0)	0 (0.0)	
Other	2 (0.9)	1 (0.7)	1 (0.4)	2 (2.1)	
Body-mass index, kg/m^2^, mean ± SD	31.1 (4.5)	29.5 (4.4)	30.6 (4.7)	31.1 (4.5)	0.01
Smoker (%)					0.73
Current	23 (10.0)	10 (7.1)	22 (9.6)	9 (9.6)	
Never	123 (53.7)	86 (61.4)	130 (57.0)	48 (51.1)	
Previous	83 (36.2)	44 (31.4)	76 (33.3)	37 (39.4)	
**Vital signs at screening**					
Systolic blood pressure, mmHg, mean ± SD	133 (9)	116 (8)	134 (8)	127 (10)	< 0.001
Diastolic blood pressure, mmHg, mean ± SD	79 (7)	69 (8)	78 (7)	74 (8)	< 0.001
Heart rate, beats/minute, mean ± SD	72 (12)	67 (10)	67 (10)	69 (10)	< 0.001
**Medical history, N, %**					
Hypertension	185 (80.8)	99 (70.7)	167 (73.2)	72 (76.6)	0.12
Dyslipidemia	40 (17.5)	23 (16.4)	37 (16.2)	17 (18.1)	0.97
Duration of T2DM, years, mean ± SD	14.4 (10.1)	14.8 (12.2)	13.8 (8.7)	15.4 (9.7)	0.96
**Concomitant medications, N, %**					
Statins	180 (78.6)	119 (85.0)	180 (78.9)	79 (84.0)	0.33
ACEi or ARB	181 (79.0)	103 (73.6)	164 (71.9)	71 (75.5)	0.35
Beta blocker	47 (20.5)	26 (18.6)	64 (28.1)	24 (25.5)	0.12
MRA	9 (3.9)	4 (2.9)	6 (2.6)	2 (2.1)	0.80
Hydrochlorothiazide	49 (21.4)	25 (17.9)	37 (16.2)	22 (23.4)	0.36
SGLT2 inhibitor	77 (33.6)	54 (38.6)	67 (29.4)	23 (24.5)	0.10
GLP-1 receptor agonist	58 (25.3)	48 (34.3)	49 (21.5)	18 (19.1)	0.02
Metformin	169 (73.8)	105 (75.0)	170 (74.6)	68 (72.3)	0.97
Insulin	70 (30.6)	41 (29.3)	51 (22.4)	26 (27.7)	0.23
Sulfonylurea	55 (24.0)	34 (24.3)	51 (22.4)	20 (21.3)	0.93
DPP4 inhibitor	26 (11.4)	22 (15.7)	25 (11.0)	12 (12.8)	0.55
**Laboratory tests**					
NT-proBNP, ng/L, median (Q1, Q3)	65 (32, 122)	63 (31, 109)	84 (47, 143)	82 (37, 149)	0.01
Hs-cTnT, ng/L, median (Q1, Q3)	9 (6, 12)	8 (5, 10)	9 (6, 12)	10 (7, 14)	0.02
HbA1c, mean ± SD	6.98 (0.79)	7.01 (0.73)	6.94 (0.84)	7.01 (0.74)	0.83
Hemoglobin, g/L, mean ± SD	13.8 (1.44)	13.5 (1.30)	13.7 (1.40)	13.5 (1.39)	0.09
CKD-Epi, eGFR, mL/min/1.73m^2^, mean ± SD	82.7 (16.1)	81.5 (15.2)	78.6 (16.7)	78.1 (16.5)	0.02
Albumin/Creatinine, median (Q1, Q3)	16 (9, 52)	10 (6, 23)	16 (9, 44)	14 (7, 38)	< 0.001
Total cholesterol, mg/dL, median (Q1, Q3)	152 (127, 178)	152 (132, 181)	157 (138, 186)	156 (134, 182)	0.24
HDL cholesterol, mg/dL, median (Q1, Q3)	45 (39, 57)	49 (39, 61)	49 (39, 60)	47 (38, 57)	0.17
LDL cholesterol, mg/dL, median (Q1, Q3)	71 (53, 98)	69 (57, 94)	74 (57, 97)	73 (55, 90)	0.75
Triglycerides, mg/dL, median (Q1, Q3)	116 (90, 196)	127 (105, 216)	127 (113, 149)	138 (122, 147)	0.93
**Echocardiographic measurements**					
E/e’, median (Q1, Q3)	11.3 (10.4, 13.2)	8.9 (8.0, 10.2)	7.6 (6.7, 8.6)	15.4 (10.0, 16.4)	< 0.001
GLS, %, median (Q1, Q3)	-17.7 (-19.4, -15.1)	-17.3 (-19.7, -15.2)	-18.0 (-19.3, -15.3)	-17.9 (-19.5, -15.6)	0.83
LAVI, mL/m^2^, median (Q1, Q3)	28.8 (26.0, 33.1)	22.3 (19.9, 25.4)	18.6 (16.4, 21.3)	30.2 (23.1, 38.9)	< 0.001
LVEF, %, median (Q1, Q3)	62 (59, 66]	62 (59, 66)	62 (59, 66)	63 (59, 66)	0.99
LVMI, g/m^2^, median (Q1, Q3)	83 (78, 90)	69 (64, 75)	61 (54, 67)	115 (106, 121)	< 0.001
RVSP, mmHg, median (Q1, Q3)	23 (18, 27)	23 (18, 27)	24 (20, 28)	23 (19, 28)	0.45
**Abnormal echocardiogram, N, %**					
GLS > -16%	60 (26.2)	43 (30.7)	56 (24.6)	16 (17.0)	0.13
E/e’ ≥ 13	66 (28.8)	5 (3.6)	1 (0.4)	50 (53.2)	< 0.001
LAVi > 34 mL/m^2^	47 (20.5)	3 (2.1)	2 (0.9)	30 (31.9)	< 0.001
LVMI ≥ 115 g/m^2^ in men and ≥ 95 g/m^2^ in women	10 (4.4)	0 (0.0)	0 (0.0)	72 (76.6)	< 0.001
RVSP > 35 mmHg	5 (2.2)	9 (6.4)	9 (3.9)	4 (4.3)	0.24
**Questionnaires**					
KCCQ physical limitation score, mean ± SD	89 (17)	90 (16)	89 (16)	88 (17)	0.89
KCCQ symptom stability score, mean ± SD	52 (10)	51 (8)	52 (11)	52 (11)	0.39
KCCQ symptom burden score, mean ± SD	93 (14)	92 (15)	92 (14)	92 (14)	0.96
KCCQ self-efficacy score, mean ± SD	87 (22)	84 (24)	84 (24)	83 (23)	0.41
KCCQ quality of life score, mean ± SD	87 (20)	87 (20)	88 (19)	86 (19)	0.95
KCCQ social limitation score, mean ± SD	93 (17)	93 (18)	92 (18)	92 (15)	0.94
KCCQ total symptom score, mean ± SD	93 (14)	92 (15)	92 (14)	91 (15)	0.88
KCCQ clinical summary score, mean ± SD	91 (14)	91 (14)	91 (14)	89 (15)	0.78
KCCQ overall summary score, mean ± SD	90 (15)	90 (15)	90 (15)	89 (14)	0.92
PASE score, mean ± SD	158 (94)	159 (88)	156 (89)	139 (85)	0.37
**CPET**					
Duration of CPET test, min, mean ± SD	9.91 (2.50)	9.73 (2.15)	9.78 (2.35)	9.34 (2.63)	0.30
Peak VO_2_, mL/kg/min, mean ± SD	15.9 (4.1)	15.6 (3.5)	15.9 (3.7)	15.0 (3.9)	0.24
VE/VCO_2_ slope, mean ± SD	30.9 (5.1)	31.2 (5.0)	31.5 (6.0)	31.4 (5.2)	0.74
Peak respiratory exchange rate, mean ± SD	1.17 (0.09)	1.21 (0.10)	1.17 (0.10)	1.18 (0.10)	0.002

SD denotes: standard deviation; mmHg denotes: millimeters of mercury; Kg/m^2^ denotes: kilograms per meter squared; T2DM denotes: type 2 diabetes mellitus; ACE denotes: angiotensin converting enzyme; ARB denotes: angiotensin II receptor blocker; Q denotes: quartile; ng/L denotes: nanograms per liter; g/dL denotes: grams per deciliter; eGFR denotes: estimated glomerular filtration rate; mL/min/1.73m^2^ denotes: milliliters per minute per 1.73 m squared; mg/dL denotes: milligrams per deciliter; E/e’ denotes: ratio of early transmitral Doppler filling velocity to early septal annular diastolic velocity; mL/m^2^ denotes: milliliters per meter squared; g/m^2^ denotes: grams per meter squared; KCCQ denotes: Kansas City Cardiomyopathy Questionnaire; PASE denotes: Physical Activity Scale in the Elderly; VO_2_ denotes: oxygen consumption; mL/Kg/min denotes: milliliters per kilogram per minute; VE/VCO_2_ denotes: slope of minute ventilation to carbon dioxide production

## Discussion

This study provides a comprehensive description of individuals with features of Stage B HF and abnormal exercise capacity. The detailed characteristics, results of laboratory and imaging testing, questionnaires of health status as well as baseline measures of exercise capacity from ARISE-HF help to further establish the diagnosis. In this analysis, we have shown that individuals enrolled in the ARISE-HF trial represent a unique long-standing T2DM population. Although by trial design they had adequate glucose control at the time of study entry, they otherwise had multiple high-risk features, including prevalent elevation of prognostic biomarkers, frequent abnormalities on echocardiography, and a substantial reduction in activity and exercise capacity despite a lack of a diagnosis of HF and health status scores that are strikingly normal. We also show that using clustering techniques, distinct phenogroups within the diagnosis of DbCM may be identified. To our knowledge this report is among the largest descriptions of the clinical aspects and testing results from a large cohort of individuals with features of DbCM.

The diagnosis of DbCM is a form of Stage B HF (also known as “pre-HF”) [5, 6], presumably identifying persons eligible for intervention to prevent progression to stages of overt HF. However, despite increasingly emphasized importance of HF prevention among individuals with DM [26], a comprehensive clinical description of individuals with features of DbCM remains an unmet need. The data from this analysis provide important insights into the general clinical profile of patients with features of DbCM and allow for comparisons to the previous reports of the diagnosis of DbCM, generally with smaller patient populations. Compared to the report of Wang and colleagues [10], the study participants in ARISE-HF are slightly younger without the variable of significant hyperglycemia and have less evidence for diastolic abnormalities or LV hypertrophy on imaging. This suggests heterogeneity may exist in the diagnosis of DbCM; this possibility is supported by the cluster analysis from ARISE-HF. These results set an important foundation for planned detailed analyses of the baseline characteristics of these study participants, including echocardiographic patterns, differences between the race/ethnicity and sex, biomarker analyses, as well as a more comprehensive analysis of CPET results at baseline. All of these will inform understanding of the effects of AT-001, a novel highly-selective aldose reductase inhibitor being evaluated in the ARISE-HF Trial.

Per protocol, study participants were required to have abnormal exercise capacity. Effort intolerance and reduced VO2 peak are highly prevalent conditions in uncomplicated, otherwise asymptomatic T2DM; this is frequently associated not only with myocardial dysfunction but abnormalities of skeletal muscle oxygen extraction [27]. The substantial reduction in exercise capacity in tandem with reduced activity levels but rather normal health status results in the various KCCQ domains, suggests “adjustment” in the daily activity levels of affected individuals. This finding supports the recent recommendation from the American Diabetes Association to diligently screen individuals for Stage B HF whenever possible [26], in an effort to initiate treatments proven to prevent progression to more symptomatic stages of the disease. Understanding the clinical presentation of the diagnosis is a critical first step.

Clinically, duration and significance of hyperglycemia has been suggested as a potent risk factor for DbCM as most individuals with the diagnosis have been reported to have more chronic diagnoses of T2DM [6, 10, 28, 29]. The individuals in ARISE-HF did have advanced age and prolonged history of T2DM. In an effort to further establish the features of DbCM, this study provides further clinical characterization, imaging and biomarker results, and the outcomes from health status and activity questionnaires.

An important aspect of this work is the demonstration of various phenogroups using cluster methodology; in this regard, we identified different phenotypic categories within the overall diagnosis of DbCM with variable distribution of clinical variables and risk factors for HF. Although impaired exercise capacity was an inclusion criterion for the ARISE-HF trial one might assume these groups might have differing levels of exercise impairment but this was not the case: despite heterogeneity of clinical presentation and different PASE scores, each of these phenogroups had comparable impairment of exercise capacity on CPET. Nonetheless, obvious clinical heterogeneity was present between the identified clusters ranging from a lower risk, younger population with normal blood pressures and lower BMI to a high risk cluster that generally resembles the original description by Rubler et al [1], with most advanced age, worst kidney function, most stigmata of diastolic dysfunction (with elevated E/e’ and highest LAVi) and greatest increase in LV mass among the study participants in this trial. This mix of patient presentations might imply differences in disease stage and might inform different diagnostic approaches for those with possible DbCM. The clinical and pathophysiologic relevance of the clusters requires further study.

Limitations of this analysis include the fact it is mainly descriptive, detailing characteristics of a population of patients that—by protocol design—lacks other risk factors for HF that are common in T2DM, such as poorly controlled glucose and/or blood pressure, advanced kidney disease, or coronary artery disease. While all these other factors no doubt contribute to and worsen risk for HF in those with DbCM, their exclusion was an expectation of regulators to allow the proper assessment of the intrinsic effects of AT-001. Future studies will need to consider the means by which DbCM will be identified in a less restrictive patient population. Although overt ischemic heart disease was an exclusion criterion, we did not perform stress testing prior to study enrollment; evidence for severe coronary ischemia would have been detected on baseline CPET. Additionally, while globally recruited and with equal sex balance and reasonable racial distribution, more data in higher risk populations such as Black individuals will be needed from future studies. As well, longitudinal results for biomarkers and other variables are not currently available as the ARISE-HF trial is ongoing; these data will be available subsequent to study completion. Nonetheless, these baseline data set an important starting point for all analyses from the trial. Although the cluster analysis provides a unique perspective on the potential clinical heterogeneity of DbCM, clustering approaches without relevant variables may lack accuracy. Lastly, as cardiac magnetic resonance imaging was not performed in this relative larger, global trial, other mechanisms associated with reduced exercise capacity in T2DM such as presence of epicardial adipose tissue [30], myocardial triglyceride content [31] or presence and extent of myocardial fibrosis [32] were not explored in ARISE-HF.

## Conclusions

In conclusion this study provides the baseline characteristics of the study population of the ARISE-HF Trial, a group of patients with T2DM, Stage B HF, and features of DbCM. Many of these individuals have evidence for risk to progression to symptomatic stages of the diagnosis with features such as elevated biomarkers and abnormal echocardiograms (or both). These baseline data from the ARISE-HF Trial will provide important information to assist in recognition of DbCM in clinical practice [26]. More information regarding heterogeneity within the overall diagnosis of DbCM is needed and efficient strategies to identify DbCM will be needed; this may include a combination of clinical variables and biomarkers together with imaging [9, 10, 33]. Furthermore, these high-level data now provide an important foundation for more detailed analyses of various aspects in the baseline results and set the stage for the read out of the efficacy and safety results of AT-001 in the ARISE-HF Trial.

### Electronic supplementary material

Below is the link to the electronic supplementary material.

Supplementary Material 1

## Data Availability

The datasets generated and/or analysed during the current study are not publicly available due to the ongoing ARISE-HF trial but are available from the corresponding author on reasonable request following completion of the study.

## Abbreviations

ARISE-HF: Aldose Reductase Inhibition for Stabilization of Exercise Capacity in Heart Failure
T2DM: Type 2 diabetes mellitus
DbCM: Diabetic cardiomyopathy
CAD: Coronary artery disease
HF: Heart failure
KCCQ: Kansas City Cardiomyopathy Questionnaire
PASE: Physical Activity Scale of the Elderly
CPET: Cardiopulmonary exercise testing
NT-proBNP: N-terminal pro-B type natriuretic peptide
hs-cTnT: High sensitivity cardiac troponin T
LVEF: Left ventricular ejection fraction
GLS: Global longitudinal strain
LAVi: Left atrial volume index
LVMi: Left ventricular mass index
RVSP: Right ventricular systolic pressure
