# Supplementary material for: Characterizing diabetic cardiomyopathy: baseline results from the ARISE-HF trial
Source: Cardiovasc Diabetol. 2024 Feb 1;23:49. doi: 10.1186/s12933-024-02135-z (PMC10835978; doi:10.1186/s12933-024-02135-z)
Supplement: Supplementary file 1 — Supplementary Material 1 [file 12933_2024_2135_MOESM1_ESM.docx]

**Characterizing Diabetic Cardiomyopathy: Baseline Results from the ARISE-HF Trial**

**Supplemental Appendix**

**Supplemental Figure 1:** Countries participating in the ARISE-HF Trial and enrollment statistics.

**
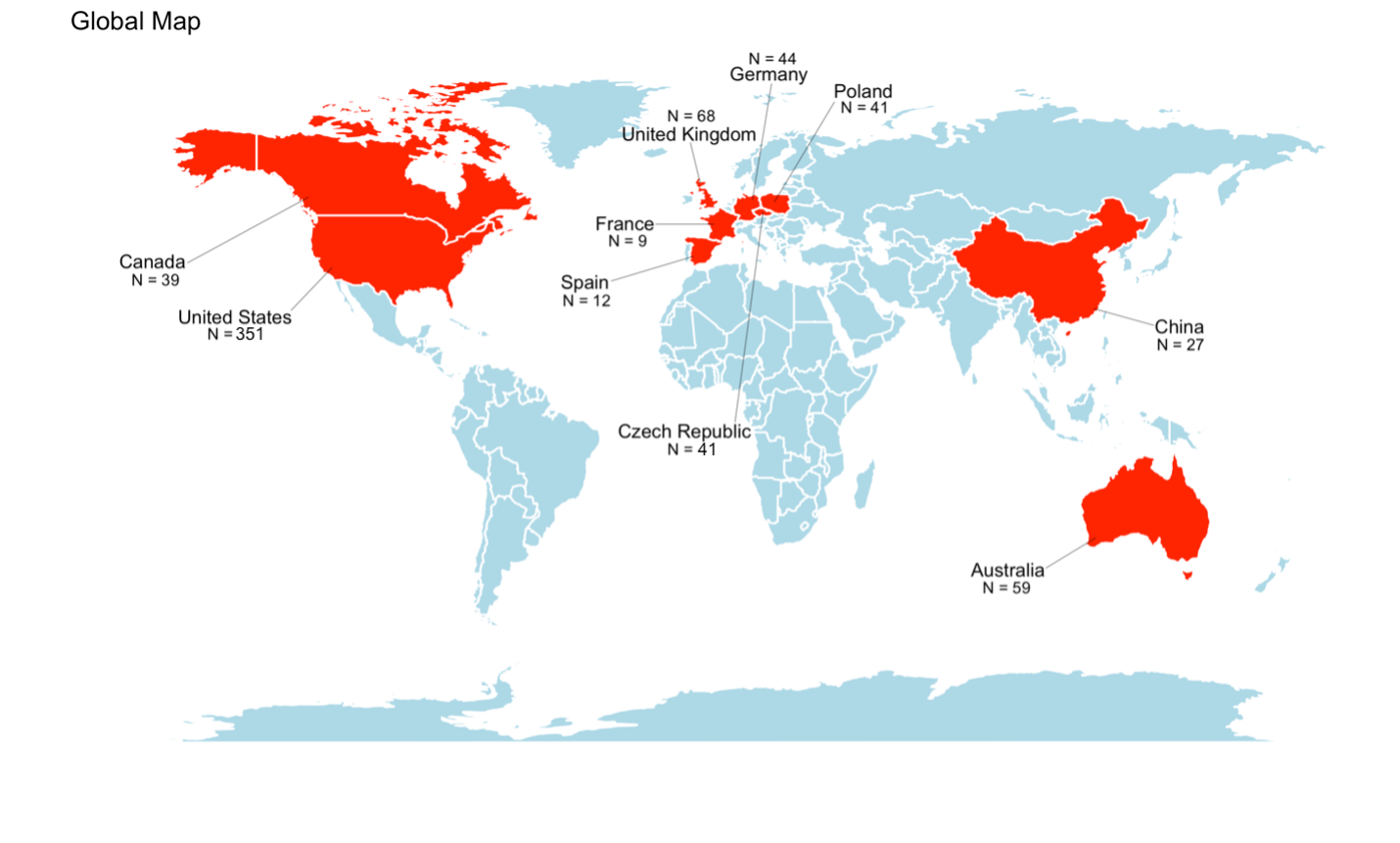
**

**Supplemental Table 1:** Key inclusion/exclusion criteria for the ARISE-HF Trial.

| **Inclusion Criteria** | **Exclusion Criteria** |
| --- | --- |
| - Diagnosis of T2DM - High risk for HF including advanced age, longstanding T2DM or CKD - Demonstration of DbCM with at least one of the following:   - Structural heart disease on echocardiography^*^   - NT-proBNP ≥ 50 ng/L   - hs-cTnT ≥ 6 ng/L - Impaired exercise tolerance on cardiopulmonary exercise testing, with peak oxygen uptake <75% of predicted and adequate exercise effort with a respiratory exchange ratio ≥ 1.05 | - Past or present diagnosis of symptomatic (stage C/D) HF or other forms of heart muscle disease - Past or present LVEF <40% - Past or present use of loop diuretics - Prior acute coronary syndrome or stroke - Significant coronary artery disease with or without revascularization - Severe valvular heart disease - Recent cardiac arrhythmia - Uncontrolled hypertension - Hemoglobin A1c > 8.5% - Hemoglobin < 10.0 g/dL - eGFR < 45 mL/min/1.73m^2^ - Body-mass index ≥ 45 Kg/m^2^ - Inability to exercise - Recurrent kidney stones |

T2DM denotes: type 2 diabetes mellitus; HF denotes: heart failure; CKD denotes: chronic kidney disease; DbCM denotes: diabetic cardiomyopathy; LVEF denotes: left ventricular ejection fraction; g/dL denotes: grams/deciliter; eGFR denotes: estimated glomerular filtration rate; mL/min/1.73m^2^ denotes: milliliters/minute/1.73 squared meters; Kg/m^2^ denotes: kilograms per squared meter

**Supplemental Table 2**: A comparison of selected baseline characteristics of those patients not included versus those included in the ARISE-HF Trial.

|  | **Excluded** | **Included** | **p** |
| --- | --- | --- | --- |
| n | 1416 | 691 |  |
| Age, mean (SD) | 66.8 (8.2) | 67.4 (7.2) | 0.12 |
| Female sex, N (%) | 670 (47.3) | 348 (50.4) | 0.21 |
| Race, N (%) |  |  | 0.15 |
| White, non-Hispanic | 877 (61.9) | 432 (62.5) |  |
| Hispanic | 294 (20.8) | 151 (21.9) |  |
| Black | 119 (8.4) | 42 (6.1) |  |
| Asian | 109 (7.7) | 56 (8.1) |  |
| American Indian or Alaskan Native | 2 (0.1) | 4 (0.6) |  |
| Other | 15 (1.1) | 6 (0.9) |  |
| Smoking, N (%) |  |  | 0.81 |
| Current | 135 (9.6) | 64 (9.3) |  |
| Previous | 470 (33.3) | 240 (34.7) |  |
| Never | 806 (57.1) | 387 (56.0) |  |
| Duration of T2DM, years, mean (SD) | 14.2 (8.3) | 14.5 (10.1) | 0.75 |

SD denotes: standard deviation, T2DM denotes: type 2 diabetes mellitus
